# Integration of monolithic porous polymer with droplet-based microfluidics on a chip for nano/picoliter volume sample analysis

**DOI:** 10.1186/s40580-014-0003-9

**Published:** 2014-03-28

**Authors:** Jin-young Kim, Soo-Ik Chang, Andrew J deMello, Danny O’Hare

**Affiliations:** 1 Department of Biosystems Science and Engineering, Bioegineering Laboratory, ETH Zurich, CH-4058 Basel, Switzerland; 2 Department of Biochemistry, Chungbuk National University, Cheongju, Chungbuk 361-763 Korea; 3 Institute of Chemical and Bioengineering, Department of Chemistry and Applied Bioscience, ETH Zurich, CH-8093 Zurich, Switzerland; 4 Department of Bioengineering, Imperial College London, London, SW7 2AZ UK

## Abstract

In this paper, a porous polymer nanostructure has been integrated with droplet-based microfluidics in a single planar format. Monolithic porous polymer (MPP) was formed selectively within a microfluidic channel. The resulting analyte bands were sequentially comartmentalised into droplets. This device reduces band broadening and the effects of post-column dead volume by the combination of the two techniques. Moreover it offers the precise control of nano/picoliter volume samples.

## Background

Microfabricated systems have emerged as promising tools for chemical and biological analysis due to the speed, throughput and control of minute samples that they make possible [1–8]. In recent years, there has been considerable interest in droplet-based microfluidics because flow segmentation enables compartmentalisation of reagent volumes from fL to μL within a continuous and immiscible fluid, as well as the generation of monodisperse droplets and the precise control of them [9–11].

MPP potentially offers the advantages of simple control of permeability and surface areas as well as easy preparation within a micro-fluidic channel for various applications of lab-on-a-chip (LOC) [12–15]. Various trials have been conducted to combine separation and droplet functions on a chip using beads-packed channels or capillary electrophoresis (CE) separation [16–20]. However, integration of MPP with droplet microfluidics has not been reported yet. Integration of MPP with droplet-based microfluidics on a single chip will provide various benefits. For example, MPP can be used as a nanostructure for a column or membrane to separate and filter analytes in the microfluidic channel. Subsequent compartmentalisation by droplets following MPP dramatically reduces Taylor dispersion of the effluent and minimizes dead volume effects; in addition, it allows further analysis downstream or offline with the encapsulated analytes. Furthermore, it is easier (in comparison to the widely-used beads) to form a porous nanostructure in a channel using selective UV polymerization and to control properties such as permeability or porosity by adjusting the composition of the MPP solution.

Our device consists of two parts: an MPP-filled channel and a droplet-generation zone. Thermoset polyester (TPE) was chosen as a device material because of its excellent mechanical properties, allowing it to withstand pressures of up to 18 Mpa [21]. It makes possible a broad range of device applications including membrane, separation column and high-frequency droplet generation; these require operational stability under high pressure, which is not feasible when using typical materials, such as polydimethylsiloxane (PDMS) for the microfluidic device.

## Methods

### Materials

TPE (CFS Fibreglass, UK) was prepared by mixing it with its polymerization catalyst, methyl ethyl ketone peroxide (MEKP), and polyethylene terephthalate, PET, (Daedong Polymer, South Korea) was used as substrate. The mixture of ethylene diacrylate (EDA, monomer) 0.485 g, methyl methacrylate (MMA, cross-linker) 0.485 g and benzophenone (BP, photo-initiator) 0.03 g was prepared for the grafting layer. Butyl methacrylate (BuMA, monomer) 0.6 g, ethylene dimethacrylate (EDMA, cross-linker) 0.4 g, 1-dodecanol (progen) 1.5 g and 2,2’-dimethoxy-2-phenylacetophenone (DMPAP, photo-initiator) 0.01 g were mixed for the monolithic porous polymer.

For droplet experiments, a 10% (v/v) mixture of fluorocarbon oil, FC-70 (3 M Fluorinert, USA), and 1H, 1H, 2H, 2H-perfluorooctanol (PFO, Sigma-Aldrich) was used for the continuous phase. The mobile phase was a mixture of 26 mM phosphate buffer (pH 7) and methanol (HiPerSolve for HPLC, BDH Prolabo) in a ratio of 5: 95 (v/v)

Fluorescein isothiocyanate, FITC, (Sigma-Aldrich, USA) and Alexa Fluor® 488, AF 488, (Invitrogen, USA) were diluted to 0.02 μM and 50 μM concentration in the mobile phase as the analytes.

### Fabrication procedure

**Figure 1:**
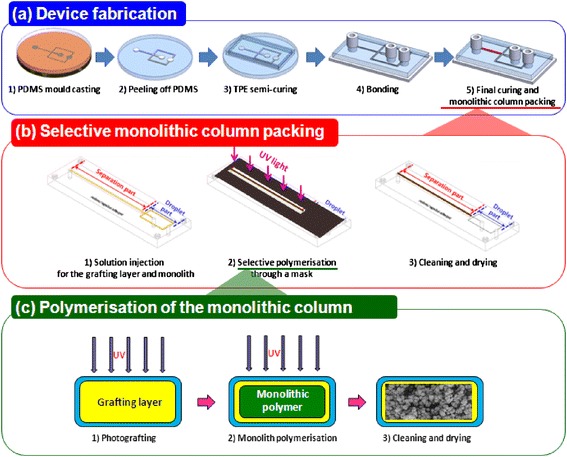
**Schematic of the hybrid monolithic LC-droplet device fabrication, (a) overall procedures - 1) PDMS mould casting from SU-8 master mould, 2) peeling of the PDMS, 3) TPE pouring and semi-curing, 4) TPE bonding to the substrate and attaching the interconnects, 5) full-curing and monolithic column packing, (b) selective packing of the monolithic column within a microchannel – 1) solutions injection, 2) selective UV exposure through a mask, 3) cleaning and drying and (c) polymerisation of the monolithic column – 1-1) injection of the grafting layer solution, 1-2) UV polymerisation through a mask, 1-3) cleaning and drying, 2-1) injection of the monolithic polymer solution, 2-2) UV polymerisation through a mask, 2-3) cleaning and drying.**

The fabrication process of the MPP-integrated droplet device is shown schematically in Figure 1. It consists of two major steps: forming the TPE microfluidic channel followed by the creation of a monolithic porous polymer. The former was made with an SU-8 master mould, fabricated using standard photolithography. PDMS was poured onto the SU-8 mould, cured for 4 h at 65°C, and then peeled off. The embossed microfluidic channel pattern on the PDMS was surrounded with 4-mm-thick walls in order to define the final outside dimensions of the device. TPE resin was mixed with the MEKP catalyst in a ratio of 100: 1 (w/w) and degassed and decanted into the PDMS mould. It was partially cured for 10 min at 60°C then cooled down to room temperature for 5 min before being separated from the PDMS mould. In the meantime, the PET substrate was cleaned by sonification in isopropyl alcohol (IPA) and was treated by O_2_ plasma at 70 mW for 12 sec in order to obtain a robust bonding. While the fully cured TPE is hard material, the semi-cured TPE is and has gel-like property. It was carefully removed and bonded to the PET substrate. To connect the microchannels with the syringe, PEEK unions (Phenomenex, USA) were placed on the inlets and outlets. The assembly was then put into a vacuum desiccator to remove residual gas. Finally, the TPE device was cured at 76°C for 1 h. This two-stsge process was adopted because semi-cured TPE substrate can be easily removed from the PDMS mould but adheres and potentially damages the SU-8 master. In addition, the flexibility of the PDMS working mould greatly facilitates the disassembly of the substrate and mould.

Next, as shown in Figure 1(b) and (c), the separation zone of the channel was selectively packed with poly(methyl acrylate) MPP. The TPE channel was selectively exposed to a UV light through a film mask during the polymerisation of the grafting layer and MPP. N_2_ gas was blown for 10 min into the grafting layer mixture (See section 2.1.) before filling the channel in order to remove oxygen and avoid expansion and subsequent heat-induced voids during polymerisation. Then it was radiated to UV light (broadband 290-385 nm, 12.22 mW/cm2) for 10 mins. The channel was flushed with 10 volumes of cleaning solvent (methanol : DI water = 1:1 (v/v)) to remove the unreacted polymer and was then dried at 40C. The monolithic polymer mixture was prepared and irradiated in a similar fashion as the grafting layer. Subsequently, it was cleaned by flowing the solvent at 10ul/min for 1 h to remove the remaining porogenic solvent and photo-initiators. It was then left to dry at 40°C overnight. UV irradiation was conducted from below since PET has much higher transmittance in the UV range than TPE does.

### Characterisation

The mobile phase was introduced into the MPP-filled channel at 10 μL/min by HP 1050 HPLC pump (Agilent, USA). 5 μL of the mixture of two dyes in the mobile phase was injected; then the effluent was segmented by the oil injected at 100 μL/min using precision syringe pumps (Harvard Apparatus, USA). Droplets containing the dyes were recorded by a high speed camera (Phantom, USA) and detected by laser-induced fluorescence (LIF) detection with a beam from 488 nm Ar^+^ diode laser (Omnichrome, Melles Griot, Cambridge, UK).

## Results and discussion

**Figure 2:**
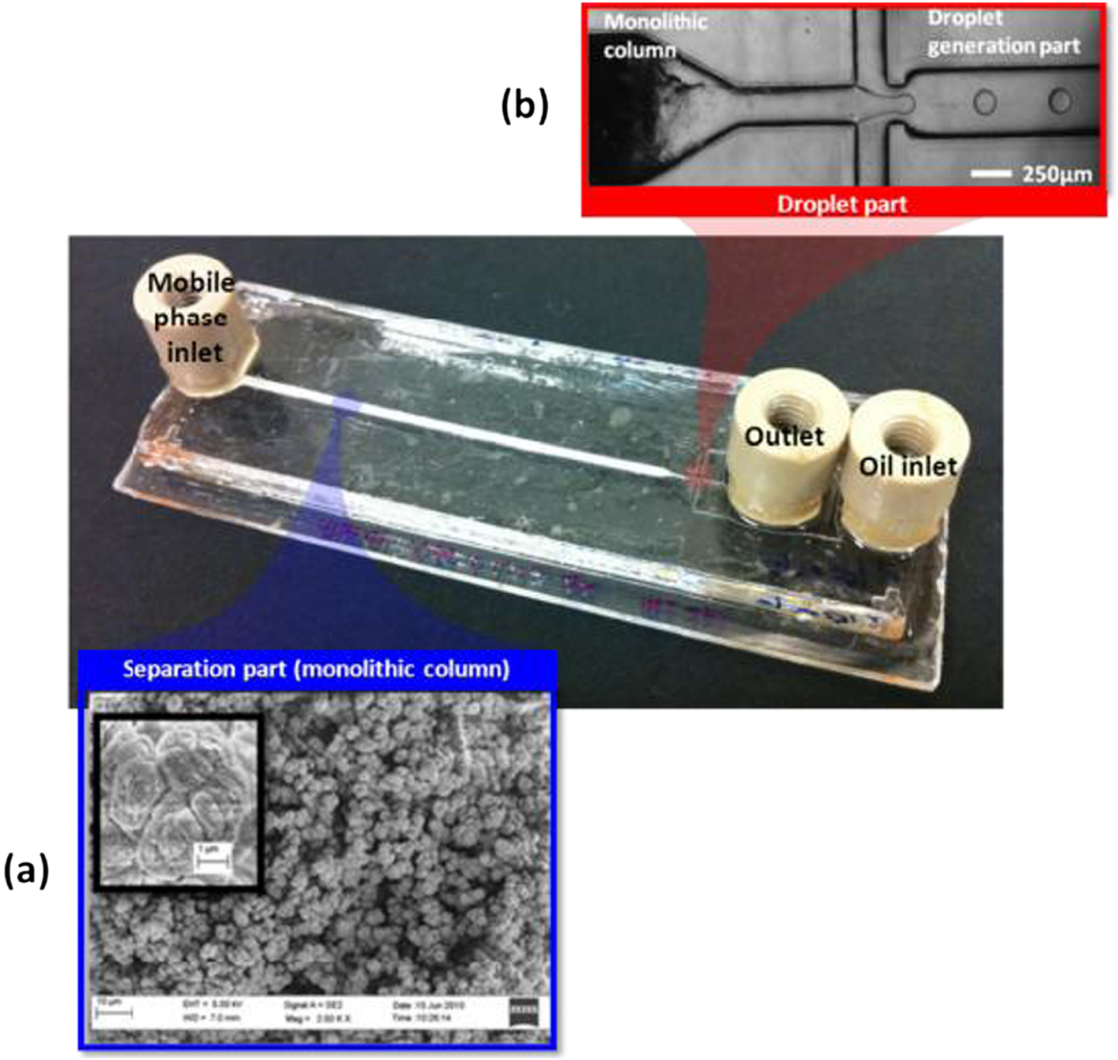
**The MPP-integrated droplet device (a) an image of the monolithic column in 1 mm (w) × 0.25 mm (H) × 50 mm (L) separation channel and (b) the 250 μm wide droplet channel.**

The fabricated MPP-integrated droplet device is illustrated in Figure 2. The poly(methyl acrylate) MPP was a selectively-formed well in the separation part only and the flow-focusing channel for droplet generation flanked the MPP within a single layer. Figure 2(a) shows SEM images of the poly(methyl acrylate) MPP within TPE channels. It consists of a number of globes that are cross-linked to each other and the globular structure includes numerous mesopores (2-50 nm) between the globes. Furthermore micropores (< 2 nm) were observed on the surface of the globes. These pores significantly contribute to increase the surface area which results in more reactions or interaction sites. Each globe is approximately 2 μm in diameter and the monolith is bonded securely to the channel wall by the grafting layer.

**Figure 3:**
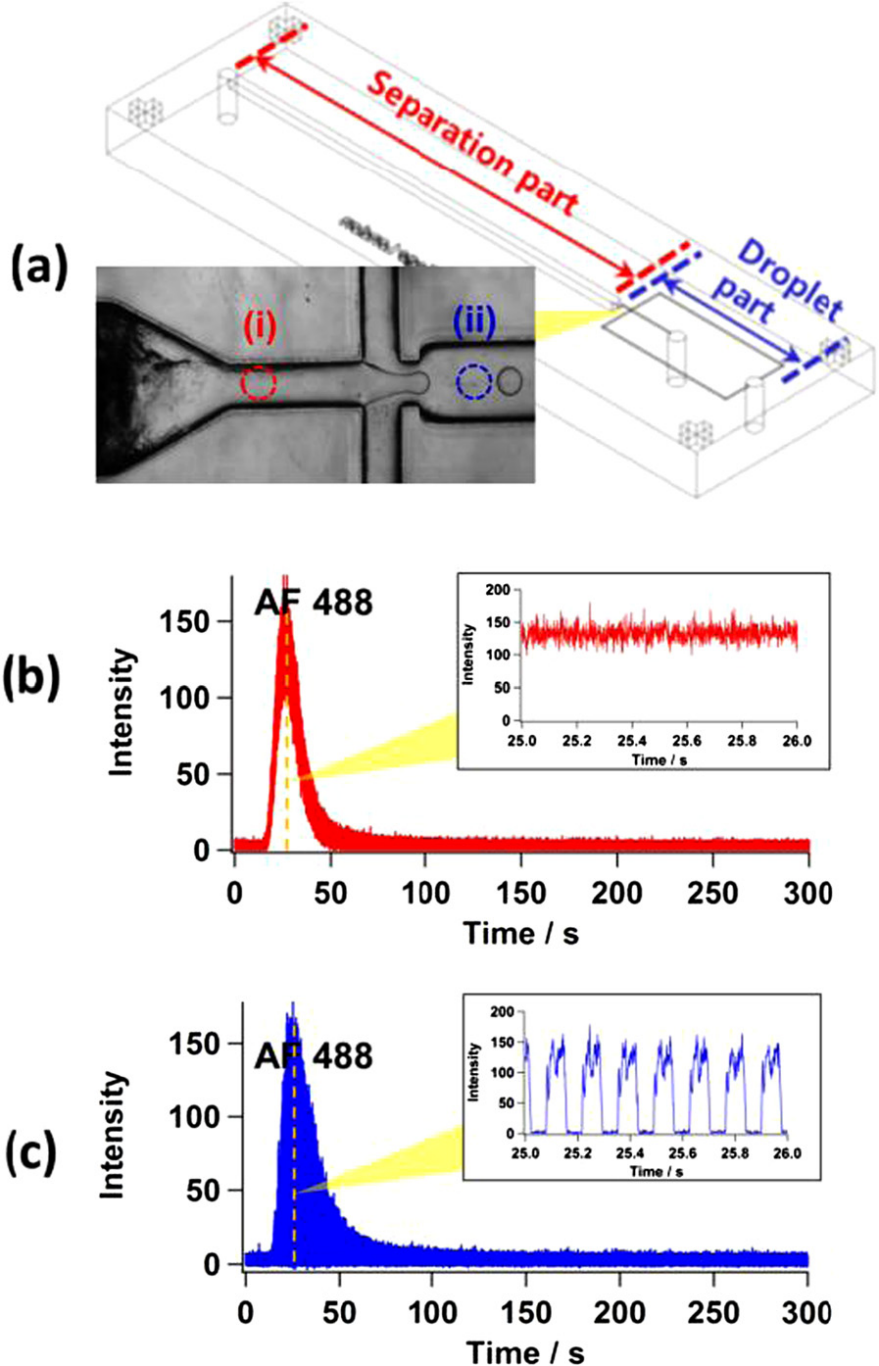
**LIF detection of AF 488 and FITC, (a) schematic of the movement of fluids in the channels and the two laser detection points, (b) the chromatograms of the separated dyes from point (і) and (c) compartmentalised dyes from point (іі).**

Two dyes, AF 488 and FITC, were injected and fluorescence was measured at two different points: before (point i) and after (point ii) the droplet generation to assess the efficacy of compartmentalisation in preserving concentration gradients, as shown in Figure 3(a). Figure 3(b) and (c) describe fluorescent peaks from the two measurement points. While the continuous signal was observed at point i, the segmented signals associated with the compartmentalised analyte by droplets are evident at point ii. No dislocation or clogging of MPP in the channel was observed during the run for over 1 hr. Moreover, since concentrations in the eluted bands are approximately Gaussian at the MPP outflow, variation in concentration between droplets can be controlled [20,22]. However separation between the dyes was poor. Only AF 488 was observed without the FITC peak, even though the measurement continued for up to 1 hr. Therefore the separation conditions for the dyes in the fabricated MPP column such as mobile phase composition and flow rate must be optimized.

To investigate diffusional band broadening, the chromatograms in (b) and (c) were quantitatively compared by extracting the maximum fluorescence of each droplet.

**Figure 4:**
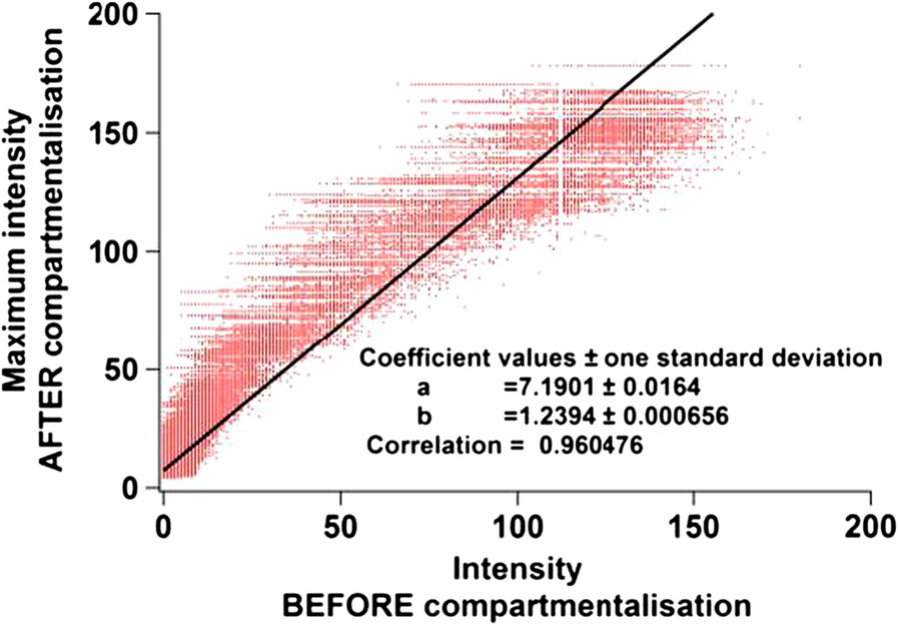
**Linear best fit between the chromatograms before (Figure** 3 **(b)) and after (Figure** 3 **(c)) compartmentalisation.**

A digital maximum filter with a 100-point window size (Igor Pro 6, USA) (slightly longer than the maximum interval between the droplets) was applied. As shown in Figure 4, the two chromatograms were strongly correlated with a slope of 1.2394 ± 0.000656 and a correlation coefficient of 0.96, which means there is no significant additional band broadening between due to compartmentalisation of the analyte and that droplet flow can act as an effective fraction collector

## Conclusions

MPP has been integrated with droplet microfluidic channel on a monolithic chip. Straightforward fabrication of monolithic porous nanostructure within a microfluidic channel has been demonstrated. Band broadening and dead volume issues have been decreased as well by sequential compartmentalisation of analytes at the MPP outflow, encapsulating them in defined droplet volumes. This confirms the potential of MPP for separation or filtering function in a microfluidic chip and the ability of droplets to act as a fraction collector for the handling of nano/picoliter volume samples.
